# Alterations of Regional Homogeneity in Children With Congenital Sensorineural Hearing Loss: A Resting-State fMRI Study

**DOI:** 10.3389/fnins.2021.678910

**Published:** 2021-10-06

**Authors:** Pingping Guo, Siyuan Lang, Muliang Jiang, Yifeng Wang, Zisan Zeng, Zuguang Wen, Yikang Liu, Bihong T. Chen

**Affiliations:** ^1^Department of Medical Ultrasound, Affiliated Tumor Hospital of Guangxi Medical University, Nanning, China; ^2^Department of Radiology, First Affiliated Hospital of Guangxi Medical University, Nanning, China; ^3^Institute of Brain and Psychological Sciences, Sichuan Normal University, Chengdu, China; ^4^Department of Radiology, Seventh Affiliated Hospital of Sun Yat-sen University, Shenzhen, China; ^5^Department of Otorhinolaryngology Head and Neck Surgery, First Affiliated Hospital of Guangxi Medical University, Nanning, China; ^6^Department of Diagnostic Radiology, City of Hope National Medical Center, Duarte, CA, United States

**Keywords:** congenital sensorineural hearing loss, kendall coefficient consistency, coherence-based regional homogeneity, functional magnetic resonance imaging, neuroimaging

## Abstract

**Background:** Brain functional alterations have been observed in children with congenital sensorineural hearing loss (CSNHL). The purpose of this study was to assess the alterations of regional homogeneity in children with CSNHL.

**Methods:** Forty-five children with CSNHL and 20 healthy controls were enrolled into this study. Brain resting-state functional MRI (rs-fMRI) for regional homogeneity including the Kendall coefficient consistency (KCC-ReHo) and the coherence-based parameter (Cohe-ReHo) was analyzed and compared between the two groups, i.e., the CSNHL group and the healthy control group.

**Results:** Compared to the healthy controls, children with CSNHL showed increased Cohe-ReHo values in left calcarine and decreased values in bilateral ventrolateral prefrontal cortex (VLPFC) and right dorsolateral prefrontal cortex (DLPFC). Children with CSNHL also had increased KCC-ReHo values in the left calcarine, cuneus, precentral gyrus, and right superior parietal lobule (SPL) and decreased values in the left VLPFC and right DLPFC. Correlations were detected between the ReHo values and age of the children with CSNHL. There were positive correlations between ReHo values in the pre-cuneus/pre-frontal cortex and age (*p* < 0.05). There were negative correlations between ReHo values in bilateral temporal lobes, fusiform gyrus, parahippocampal gyrus and precentral gyrus, and age (*p* < 0.05).

**Conclusion:** Children with CSNHL had RoHo alterations in the auditory, visual, motor, and other related brain cortices as compared to the healthy controls with normal hearing. There were significant correlations between ReHo values and age in brain regions involved in information integration and processing. Our study showed promising data using rs-fMRI ReHo parameters to assess brain functional alterations in children with CSNHL.

## Introduction

Congenital sensorineural hearing loss (CSNHL) is a disabling disease characterized by lack of sound stimulation and hearing loss at birth (Harlor and Bower, 2009; Tan et al., 2013). Hearing loss not only affects language but also causes changes to motor function and cognition (Lin et al., 2013). Cross-modal plasticity with enhanced sensory modes such as vision has been implicated in the effort to compensate for the hearing loss (Lee et al., 2001; Bavelier and Neville, 2002; Lomber et al., 2010). As a result, auditory cortex in both humans and animals with congenital deafness may remain relatively normal without degeneration or atrophy through non-sound stimulus from vision, movement, and perception (Stanton and Harrison, 2000). Nevertheless, it is unclear how brain function may be altered due to hearing loss and how brain neuroplasticity with other sensory modes may compensate for hearing deprivation in children with CSNHL.

Advanced neuroimaging has made possible for assessing *in vivo* brain structure and function (Mayer and Trezek, 2018). Resting-state functional brain MRI (rs-fMRI) is a functional imaging technique based on the blood-oxygen-level-dependent (BOLD) method to assess brain regional alterations when not performing a task (Mirzaei and Adeli, 2016). BOLD-fMRI indirectly reflects the intrinsic brain activity in the *infraslow frequency band* (< *0.1 Hz)* (Biswal et al., 1995). Compared to the task-based fMRI, rs-fMRI can detect brain functional changes and core brain networks (Buckner, 2013). *Therefore, rs-fMRI has been used to study various conditions including attention deficit disorder, social anxiety disorder, epilepsy, and blindness* (Plichta et al., 2009; Fair et al., 2010; Bedny et al., 2011; Liu et al., 2015, 2017) *for functional connectivity between the whole brain and certain preset regions of interest* (Calamante et al., 2013). However, the connectivity analysis does not usually reflect brain regional changes that are specific to the conditions under scrutiny such as hearing loss (Greicius et al., 2007). These is an unmet need to assess specific brain regions involved in hearing, vision, motor, and cognition in order to understand the effect of hearing loss on brain structure and function in children with CSNHL.

Regional homogeneity (ReHo) can be calculated based on the Kendall coefficient consistency (KCC-ReHo) from rs-fMRI, which measures the similarity of the time series of a given voxel to those of its nearest neighbors (Zang et al., 2004; Zuo et al., 2013). A prior study by Wolak et al. (2019) found that hearing development and language processing were affected by hearing loss as indicated by changes in the KCC-ReHo values. However, KCC-ReHo is known to be susceptible to random noise caused by phase delay in the time span (Ou et al., 2018). A similar method, i.e., the coherent-based ReHo (Cohe-ReHo) method, has been used to detect the regional synchronization of rs-fMRI signals. *Compared to KCC-ReHo, Cohe-ReHo is not sensitive to noise* (Liu et al., 2010) *and has been successfully utilized to detect brain regional alterations for various disorders* (Guo et al., 2012; Liu et al., 2012, 2018; Zhou et al., 2019). Therefore, both KCC-ReHo and Cohe-ReHo are useful parameters and are complimentary for assessing brain functional alterations.

In this study, we analyzed rs-fMRI for both KCC-ReHo and Cohe-ReHo to assess the differences in brain regional activity between the children with CSNHL and the healthy controls with normal hearing. We used the rs-fMRI approach because it was feasible and practical for children with CSNHL who may not be able to perform any tasks in the MRI scanner while under sedation. We hypothesized that children with CSNHL would have alterations of ReHo values, especially in specific brain regions associated with hearing, vision, motor, and executive control, when compared to the healthy controls.

## Materials and Methods

### Participants

Children with CSNHL who were right-handed and who had no treatment for hearing loss were recruited from our outpatient subspecialty clinic for hearing loss from January 2017 to October 2019. Children with normal hearing serving as healthy controls were recruited from the community. Part of this cohort was included in our previously published study of white matter microstructural analysis of diffusion tensor imaging (Jiang et al., 2019). However, we have not published any rs-fMRI data from this cohort.

Inclusion criteria included right-handed children with CSNHL diagnosed by both hearing screening tests at 3 and 42 days after birth and bilateral auditory brainstem response (ABR) > 91 db. Exclusion criteria included the following: severe neurological disorders such as epilepsy and congenital leukodystrophy, conditions with impaired cognition such as autism and severe hyperactivity syndrome, history of treatment for ear-related diseases such as infection, history of using hearing aids, and history of contraindications for MRI such as having a cardiac pacer and orbital metal. For rs-fMRI scans, all participants were sedated with 10% oral chloral hydrate in a dosage of 0.3–0.5 ml per kg of body weight with the maximal dosage of 80 ml. All children were under the care of a pediatrician or a pediatric nurse practitioner while under sedation for the brain MRI scan. No adverse effect was noted from the oral sedation in our cohort. The study was approved by the local ethics committee and institutional review board for research in our hospital. Informed consent was signed by all participants’ legal guardians.

### Resting-State Functional Brain MRI Acquisition

Rs-fMRI scans were obtained on the same Siemens Verio 3T scanner (Siemens Healthcare, Erlangen, Germany). Soft earplugs and foam pads were used to reduce the scanner noise and head motion. The rs-fMRI scans were obtained when the children were asleep after oral sedation. Gradient-echo planar imaging sequence for rs-fMRI was acquired for all participants with the following parameters: repetition time/echo time = 2,000/30 ms, 30 slices, 64 × 64 matrix, 90° flip angle, 24 cm field of view (FOV), 4 mm slice thickness, 0.4 mm gap, and 250 volumes (500 s).

### Resting-State Functional Brain MRI Data Processing

Rs-fMRI data were preprocessed with DPARSF package based on the MATLAB platform (Chao-Gan and Yu-Feng, 2010). Briefly, the first 10 volumes were discarded for signal stabilization and subject adaptation, which was followed by slice timing and spatial realignment. Subjects with excessive head motion (translational and rotational displacement exceeded 2.0 mm or 2.0°) were excluded.

All images were normalized to the standard space on the Montreal Neurological Institute (MNI) template and were resampled to 3 × 3 × 3 mm^3^. The MNI template has been known to be appropriate for normalizing brains from young children to adolescence (Chen et al., 2017, 2018). *Subsequently, linear trend, white matter signal, cerebrospinal fluid signal, and Friston 24 motion parameters were used as regressors to reduce effects of head movement and non-neuronal information* (Yan et al., 2013). Band-pass (0.01–0.08 Hz) filtering was then conducted.

*The KCC-ReHo values of the pre-defined band time series of each voxel in the entire brain relative to the nearest 27 voxels were calculated with the REST plus software*.^1^ The resulting data were further spatially smoothed using a 6-mm isotropic full width at half maximum (FWHM) Gaussian kernel to generate the ReHo map.

In addition to the KCC-ReHo values, a second set of ReHo values, i.e., the Cohe-ReHo, were calculated with the following three steps in the REST plus software (Liu et al., 2010). First, the power spectrum of time series in each voxel was estimated with the Welch’s modified periodogram averaging method. Second, the coherences between time series in each voxel and its nearest 27 voxels were estimated. Third, the 27 coherence values were averaged to represent the Cohe-ReHo of the center voxel. To eliminate the differences in the overall level of the whole brain ReHo value among the individuals, the ReHo value of each voxel was divided by the mean value of the whole brain signal amplitude. The resulting data were further spatially smoothed using a 6-mm isotropic FWHM Gaussian kernel to generate the ReHo maps for statistical analysis.

### Statistical Analysis

The demographic data were analyzed with SPSS Version 16 (SPSS, Inc., Chicago, IL, United States). Two-sample *T*-test was performed to detect the differences in ReHo values between the CSNHL group and the healthy control group using REST plus. Age, gender, and mean framewise displacement (Mean FD) were considered as covariates for estimating their effects on group difference independently. Alphasim correction was used for multiple comparisons with voxel-level significance set at *p* < 0.01 and cluster-level significance set at *p* < 0.05 (Zhou et al., 2019). We chose more lenient *p*-values for multiple comparison because of our intent to identify preliminary results for hypothesis generating, to balance α and β errors in statistical analysis, and to verify the results with two ReHo indices. All significant clusters were reported on the MNI coordinates, and the *T*-values of the peak voxel were determined.

We conducted Pearson correlation analysis between ReHo values and age with REST plus software. Focusing on the positive and negative correlation peaks, the signals in the spheres with a radius of 6 mm were extracted, and age-related scatter plots were presented.

## Results

A total of 45 children with CSNHL and 20 healthy controls were recruited into this study. The participants ranged from 0.5 to 16 years of age. There were no significant differences in gender (*p* = 0.60), age (*p* = 0.33), or head movement parameters (*p* = 0.11) between the CSNHL group and the healthy control group (Table 1).

**TABLE 1:** Participant information.

	CSNHL (*n* = 45)	HC (*n* = 20)	*p*
Age (years ± SD)	4.82 ± 2.64 (range 1.5–13)	5.66 ± 3.36 (range 0.5–16)	0.33
Gender (Male/Female)	25/20	13/7	0.60
Handedness	45 right-handed	19 right-handed	
Mean FD	0.15 ± 0.07	0.19 ± 0.10	0.11
**dB HL**			
Left ear (dB)	105.01 ± 8.13 (97–120)		
Right ear (dB)	99.91 ± 8.74 (92–120)		

*CSNHL, congenital sensorineural hearing loss; HL, hearing loss; dB, decibel; HC, healthy control; Mean FD, mean framewise displacement.*

### Regional Homogeneity Results

**FIGURE 1:**
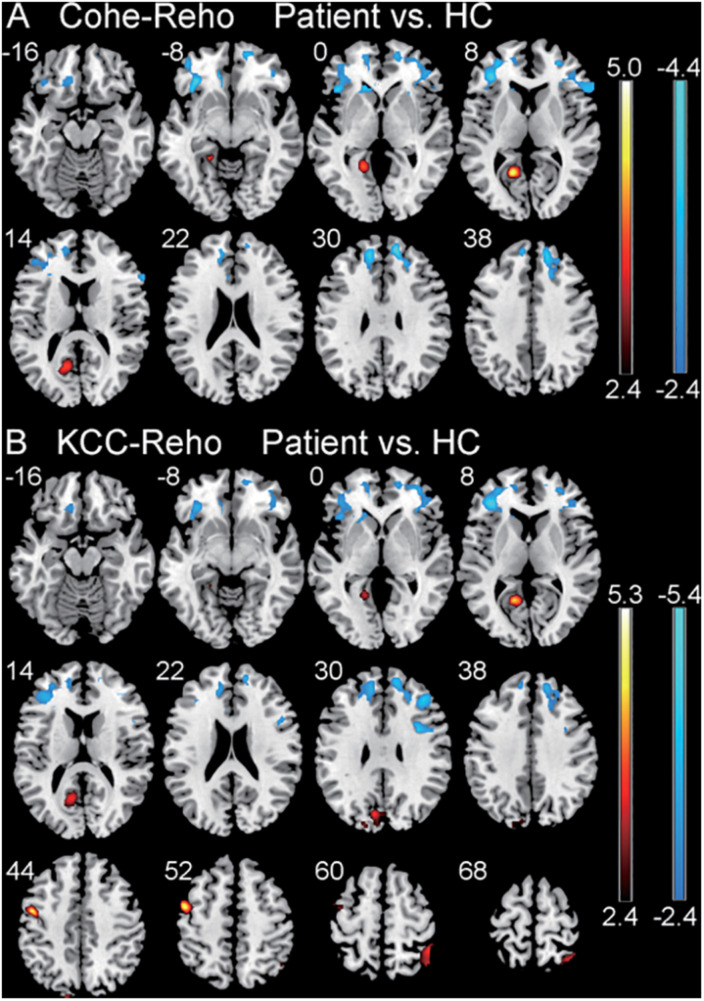
Comparison of regional homogeneity (ReHo) values between the patient group with CSNHL and the healthy control (HC) group. **(A)** Comparison of coherence-based regional homogeneity (Cohe-ReHo) values between the CSNHL group and the HC group. **(B)** Comparison of Kendall coefficient consistency (KCC-ReHo) values between the CSNHL group and the HC group.

There were significantly increased Cohe-ReHo values in left calcarine and decreased values in bilateral ventrolateral prefrontal cortex (VLPFC) and right dorsolateral prefrontal cortex (DLPFC) in the CSNHL group when compared to the healthy control group with corrected *p* < 0.05 for all clusters (Table 2 and Figure 1A).

**TABLE 2:** Differences in regional homogeneity (ReHo) values between the congenital sensorineural hearing loss (CSNHL) group and the healthy control (HC) group.

Index	Breakpoint cluster region (BCR)	Cluster size	Coordinate (mm)			*t*-value
			X	Y	Z	
Cohe-ReHo	**CSNHL > HC**					
	Left calcarine	190	–15	–57	9	4.96
	**CSNHL < HC**					
	Left VLPFC	737	–12	36	–6	–4.30
	Right VLPFC	352	30	48	6	–3.90
	Right DLPFC	222	12	51	30	–4.38
KCC-ReHo	**CSNHL > HC**					
	Left calcarine	146	–15	–57	9	4.88
	Left cuneus	164	–9	–78	30	3.62
	Left precentral	158	–48	–3	48	5.30
	Right SPL	196	48	–45	63	4.36
	**CSNHL < HC**					
	Left VLPFC	661	–36	36	9	–5.40
	Right DLPFC	702	12	54	24	–4.68

*VLPFC, ventrolateral prefrontal cortex; DLPFC, dorsolateral prefrontal cortex; SPL, superior parietal lobule. All clusters were corrected with p < 0.05.*

There were significantly increased KCC-ReHo values in the left calcarine, left cuneus, left precentral, and right superior parietal lobule (SPL) and decreased values in left VLPFC and right DLPFC in the CSNHL group when compared to the healthy control group with corrected *p* < 0.05 for all clusters (Table 2 and Figure 1B).

### Correlation Between Regional Homogeneity Values and Age

Significant correlations were detected between ReHo values in certain brain regions and the age of children with CSNHL with Alphasim- corrected *p* < 0.01 at the voxel level and *p* < 0.05 at the cluster level. There was no correlation between the ReHo values and age in the healthy control group after Alphasim correction (*p* > 0.05).

**FIGURE 2:**
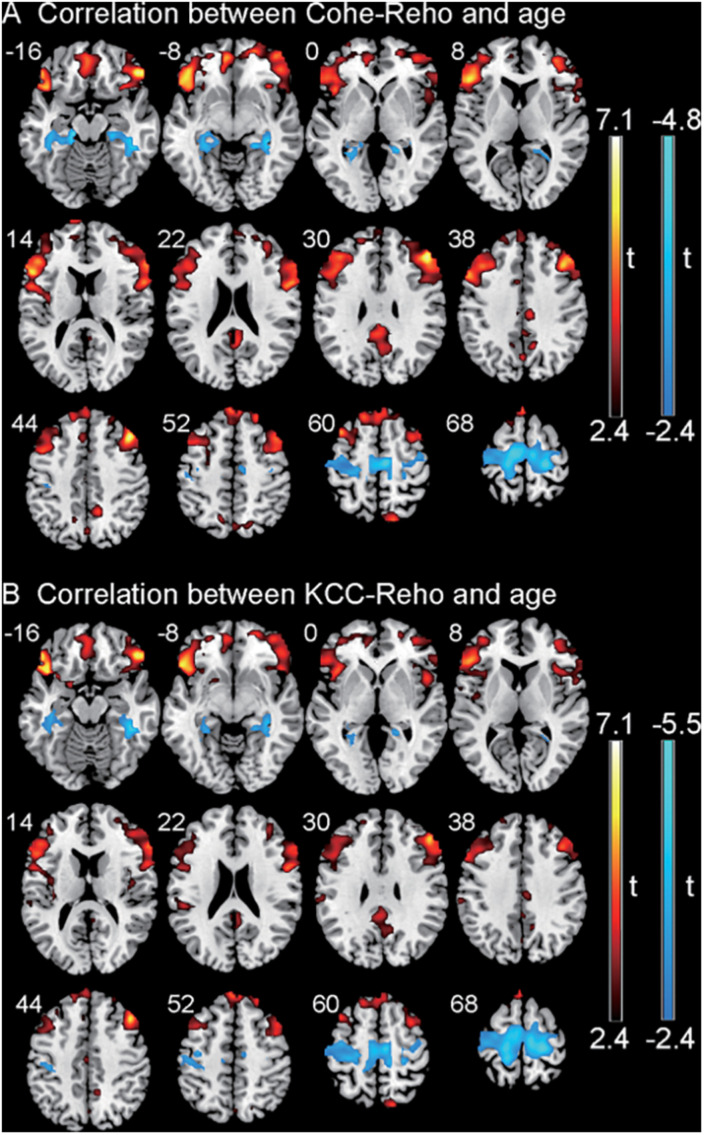
Correlation between regional homogeneity (ReHo) values and age in children with congenital sensorineural hearing loss (CSNHL). Positive correlations were indicated in red color, and negative correlations were indicated in blue color. **(A)** Correlation between coherence-based regional homogeneity (Cohe-ReHo) values and age. **(B)** Correlation between Kendall coefficient consistency (KCC-ReHo) values and age.

**FIGURE 3:**
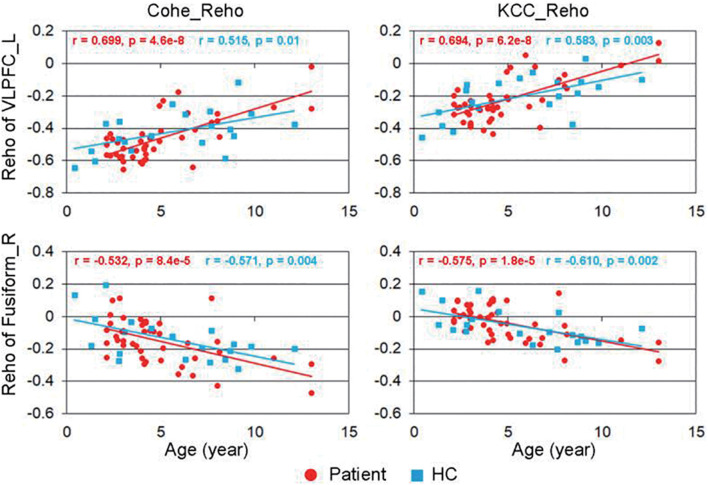
Correlation between regional homogeneity (ReHo) values and age. The strongest positive correlation was located in the right ventrolateral prefrontal cortex (VLPFC), and the most negative correlation was located in the right fusiform gyrus.

The correlation between the KCC-ReHo values and age and the correlation between the Cohe-ReHo and age were similar in the children with CSNHL. There were significant correlations between ReHo values and age in the following brain regions: prefrontal cortex (PFC), posterior cingulate cortex (PCC)/precuneus, bilateral fusiform/parahippocampal gyrus, and precentral gyrus. Among them, PFC and PCC/precuneus were positively correlated with age, with the strongest point located at the right VLPFC. Bilateral temporal lobe fusiform gyrus/parahippocampal gyrus and precentral gyrus were negatively correlated with age, with the strongest point located in the right fusiform gyrus (Table 3 and Figure 2). The mean ReHo values in each of the brain regions were extracted for both the CSNHL group and the healthy control group. Their relationships with age were presented in Figure 3.

**TABLE 3:** Correlation between regional homogeneity (ReHo) values and age in children with congenital sensorineural hearing loss (CSNHL).

Index	Breakpoint cluster region (BCR)	Cluster size	Coordinate (mm)			*t*-value
			X	Y	Z	
**Cohe-ReHo**						
	PFC	6,189	48	36	33	7.13
	PCC/precuneus	707	15	–75	60	4.81
	Right fusiform/parahippocampal	519	39	–33	–21	–4.76
	Left fusiform/parahippocampal	632	–18	–27	–18	–4.58
	Precentral gyrus	1,218	21	–27	69	–4.64
**KCC-ReHo**						
	PFC	5,230	–48	33	–12	7.11
	PCC/precuneus	449	15	–75	60	4.64
	Right fusiform/parahippocampal	401	39	–36	–18	–5.46
	Left fusiform/parahippocampal	358	–39	–21	–18	–4.58
	Precentral gyrus	1,480	18	–30	66	–5.43

*PFC, prefrontal cortex; PCC, posterior cingulate cortex: KCC-ReHo, Kendall coefficient consistency; Cohe-ReHo, coherence-based regional homogeneity.*

## Discussion

To the best of our knowledge, our study was the first to assess brain regional Cohe-ReHo alterations in children with CSNHL. We found de-synchronization of brain regional activity showing different ReHo values in different brain regions of the deaf children. In addition, we also found significant correlations between the ReHo values and age in children with CSNHL.

Our findings of increased KCC-ReHo values in left calcarine and left cuneus in the CSNHL group as compared to the healthy control group were generally in line with literature. Calcarine and cuneus as parts of occipital lobe belong to the visual cortex and are involved in visual processing. Bavelier et al. (2006) reported that deaf patients had better visual performance than those with normal hearing, especially in tasks requiring higher attention. A higher KCC-ReHo value in the right occipital lobe has been found by Xia et al. (2017) in children with CSNHL. Shiell et al. (2015) suggested that early auditory deprivation may lead to functional reorganization of the auditory cortex and may enhance interactions between auditory and visual brain regions. Converging evidence from our data and other’s observations supports the notion for cross-modal reorganization of auditory-related brain regions for the hearing impaired. Audio-visual modal reorganization is one type of the cross-modal patterns for which the deaf children may mobilize more visual resources to compensate for deprivation of sound stimuli (Bavelier et al., 2006; Xia et al., 2017).

Precentral gyrus is the primary motor cortex, and it is an important structure for autonomous movement (Rivara et al., 2003). Westermann and Reck Miranda (2004) and Shi et al. (2016) showed that infants learn language by learning the connections between sounds and actions needed for pronunciation, indicating that the synchronicity between the auditory and motor cortex may be critical in language development. For children with CSNHL, deprivation of sound and desynchronization of cortex may motivate and enhance additional sensory and motor stimuli. Therefore, it was not surprising to see our data showing increased brain activity in the motor-related cortex, i.e., precentral gyrus, which may be part of the efforts to compensate for the hearing loss.

SPL is a brain region important for cognitive and motion-related process operating as a transmitter of somatosensory, visual motor integration, and visual spatial attention (Corbetta et al., 1995; Parks and Madden, 2013). Iacoboni et al. (1999) reported that SPL could encode motion perception such as finger movements. Sign language has been used by school-age children with CSNHL for communication, and these children may activate their motor cortex through hand and finger movement (Hari et al., 1998). In line with the abovementioned studies, our results of showing increased ReHo values in the SPL may reflect the brain functional neuroplasticity to integrate the visual function and motor function through sign language.

Our study showed decreased ReHo in bilateral VLPFC and right DLPFC, which were components of the executive control network implicated in initiating and modulating cognitive function. These brain regions contain multi-sensory cells that can receive various types of afferent stimuli (Sugihara et al., 2006). Published literature has suggested that DLPFC is related to auditory spatial processing (Klaus and Schutter, 2018), while VLPFC is responsible for receiving the input of auditory cortex for non-spatial acoustic processing (Romanski et al., 1999). A prior study of amnestic mild cognitive impairment showed decreased ReHo in DLPFC and decreased local connectivity (Zhen et al., 2018). We speculated that the lack of sound stimulation may have weakened the functional integration of VLPFC and DLPFC as reflected by reduced ReHo values in these brain regions.

Our study showed both similarities and differences in the ReHo alterations between the Cohe-ReHo and the KCC-ReHo methods. For instances, both methods showed increased values in the left calcarine and decreased values in VLPFC and DLPFC. However, the KCC-ReHo method identified increased values in additional brain regions such as cuneus, precentral gyrus, and SPL, which are related to motor cortex and neuroplasticity. The mechanism underlying these results is not clear. We speculate that it might be due to the subtle variations in these two methods, such as calculation accuracy or threshold selection. Nevertheless, our pilot study identified these intriguing results, which should motivate more research to understand the implications of ReHo alterations from these two methods on functional adaptation and cross-modal plasticity for the children with hearing impairment.

We found a positive correlation between the ReHo value of PFC and PCC/precuneus with age in children with CSNHL. For our cohort, hearing deprivation became apparent at birth. Therefore, their age indicated the duration of their hearing loss and older children suffered from longer duration of hearing loss than the younger ones. We speculate that the positive correlation between ReHo and age may imply the occurrence of more brain alterations in older children due to the longer duration of their hearing loss.

Prior studies have shown that prefrontal cortex is essential in auditory cognition because it receives information from a wide range of auditory regions (Rowe et al., 2008). On the other hand, as a node in the default mode network, PCC/precuneus can simultaneously communicate with various brain networks involved in multiple brain functions such as cognition and motor (Rolls, 2019) while receiving sensory input from brain auditory regions (Rowe et al., 2008; Tanabe and Sadato, 2009). Conway et al. (2011) considered that the lack of auditory input may have reduced auditory-frontal connectivity, which may affect the development of cognitive function and motor skills for the hearing impaired. In our study, with increasing age of the children with CSNHL along with longer duration of hearing loss, we should not be surprised to see increasing ReHo values as the children grow older, thus having a positive correlation between ReHo and age. We speculate that PFC and PCC/precuneus may need stronger information integration with more brain activity to cope with disruption of the normal physiological status in some brain regions due to prolonged hearing loss.

Our study showed that the ReHo values of bilateral temporal lobe fusiform gyrus/parahippocampal gyrus and precentral gyrus were negatively correlated with age. Temporal lobe fusiform gyrus and parahippocampal gyrus participate in language decoding and semantic processing during auditory stimulus (Kravitz et al., 2011; Forseth et al., 2018). A prior study has suggested that preoperative cortical stimulation would impair the performance of reading and hearing comprehension tasks, reflecting the important role of these brain regions for completing these tasks (Binder et al., 1997). As the severity of deafness increases, these brain regions may need to increase activity to compensate for the lack of sound stimulus.

There has been extensive literature on hearing loss and rs-fMRI methodology. For instance, a study by Xia et al. (2017) enrolled infants with CSNHL and matched normal hearing controls and analyzed rs-fMRI data. They found alterations of ReHo in brain areas for language, auditory, and visual information processing in infants with CSNHL. On the other hand, our study focused on ReHo analysis with estimates of two ReHo parameters, i.e., KCC-ReHo and Cohe-ReHo. Similar to their study, we found ReHo alterations in auditory and visual brain areas. However, we also identified additional ReHo alterations in brain regions related to motor and cognitive function in children with CSNHL. Although a large range of age in our cohort was recognized as a limitation to our study, this cohort did enable us to obtain pilot ReHo data in older children beyond 4 years of age as the Xia et al. (2017) study only enrolled children up to 4 years of age.

In addition to the rs-fMRI approach, other fMRI methods have been used to study hearing loss, which has generated promising results. For example, a study by Zhang et al. (2006) used a block-design fMRI paradigm with pure tones and found differences in brain activations in subjects with sensorineural hearing loss as compared to the controls. In addition, they also found differences in audio-evoked fields on magnetoencephalography between the patient group and the control group. It should be noted that the rs-fMRI method is robust and has been used extensively to study various disorders and conditions. A study by Ni et al. (2016) used resting-state fMRI approach and found differences in ReHo values between the subjects with mild cognitive impairment with and without lacunar infarctions. Nevertheless, our study has merits and we contributed to the hearing research through assessment of the underlying brain functional neuroplasticity in children with CSNHL.

There were several limitations to this study. First, the sample size was small, which may limit our ability to detect subtle alterations in the ReHo values in our children with CSNHL. In addition, our cohort included children with a large range of age and we did not have a sufficient sample size to separate this cohort into different age groups. We understand that brain fMRI parameters may be varied with age along the course of brain development. However, we believe that this variation should be balanced off in our cohort since there was no significant difference in age between the CSNHL group and the healthy control group. Second, our analysis of brain activity did not take into consideration the severity of deafness in this cohort with CSNHL. Third, this study was cross-sectional in design, limiting our assessment for recovery or additional alterations of ReHo parameters over time. In addition, our study was limited due to our inability to incorporate education situation as a covariate in data analysis. It was because we could not definitively gauge the educational levels for our children with CSNHL due to their various educational backgrounds and socioeconomic status. Our study was also limited by other aspects of potential differences such as emotional stress between the two groups. Lastly, sign language was not considered as a confounding variable in our data analysis. We did not include the history of using sign language in our analysis because of the discrepancy in sign language use among the participants. Some children in our cohort learned and practiced sign language, while some did not. We did not have the statistical power in this small cohort to assess the effect of sign language use on the brain changes. In the future, we will enroll a larger cohort to tease out the effect of the potential confounding variables such as the severity and duration of deafness, different age groups, sign language use, and history of treatment for hearing loss on brain function.

## Conclusion

In summary, we found brain functional alterations as indicated by rs-fMRI ReHo values in the brain regions related to auditory, visual, motor, and cognitive function in children with CSNHL. We also observed a significant correlation between brain functional changes and age. Our study results implicated neuroplasticity and compensatory changes in children with CSNHL to adapt for hearing deprivation. Our study showed promising data for using an imaging approach to uncover the neural correlates of hearing loss and to improve the care of our vulnerable children with CSNHL.

## Data Availability Statement

The raw data supporting the conclusions of this article will be made available by the authors, without undue reservation.

## Ethics Statement

The studies involving human participants were reviewed and approved by the First Affiliated Hospital of Guangxi Medical University. Written informed consent to participate in this study was provided by the participants’ legal guardian/next of kin.

## Author Contributions

MJ conceived and designed the study. PG, SL, ZW, and YL collected the data. YW, PG, and SL contributed to data analysis. MJ, PG, and SL prepared the first draft of the manuscript. BC, MJ, YW, and ZZ revised the manuscript. All authors approved the final version of the manuscript.

## Conflict of Interest

The authors declare that the research was conducted in the absence of any commercial or financial relationships that could be construed as a potential conflict of interest.

## Publisher’s Note

All claims expressed in this article are solely those of the authors and do not necessarily represent those of their affiliated organizations, or those of the publisher, the editors and the reviewers. Any product that may be evaluated in this article, or claim that may be made by its manufacturer, is not guaranteed or endorsed by the publisher.

## Footnotes

1
http://restfmri.net/forum/restplus
